# A practical approach example to measurement uncertainty: Evaluation of 26 immunoassay parameters

**DOI:** 10.11613/BM.2022.030705

**Published:** 2022-08-05

**Authors:** Rabia Tan, Mustafa Yilmaz, Yusuf Kurtulmuş

**Affiliations:** 1 Department of Clinical Biochemistry, Aydın Public Health Laboratory, Aydın, Turkey; 2 Department of Medical Biochemistry, Aydın Adnan Menderes University Faculty of Medicine, Aydın, Turkey; 3 Department of Addiction Toxicology, Institute of Substance Abuse Toxicology and Pharmaceutical Sciences, Ege University, İzmir, Turkey

**Keywords:** immunoassay, quality control, uncertainty

## Abstract

**Introduction:**

Measurement uncertainty is a non-negative parameter that characterizes the distribution of all values appropriate to the measured size and is associated with the measured result. In this study, we aimed to compare the results with various suggestions and produce more qualified results by calculating the measurement uncertainties of the immunoassays like fertility hormones, drug concentration tests, cardiac markers, thyroid function tests and tumour markers.

**Materials and methods:**

Uncertainty calculation was made in accordance with the top-down approach according to Nordtest guide. The 12-month study of internal and external quality assessment results were used. The parameters of drug concentration tests were performed on the Abbott Architect c8000, other hormones/markers on the i2000 of the same brand.

**Results:**

Factors that increased the measurement uncertainty of a test were due to external quality control data. The calculations showed that 13 of 26 parameters satisfied quality requirements. The highest uncertainty value, with 28% belonged to cancer antigen 19-9 test. The lowest value was calculated for prolactin with 8.3%. Dehydroepiandrosterone sulfate and phenytoin performed poorly in terms of measurement uncertainty, although internal and external quality control assessment results were considered favourable for both.

**Conclusion:**

It is recommended that the concept of measurement uncertainty, which plays an important role in the total quality performance of the laboratory, should be followed up by the clinical laboratory experts at certain time intervals and should be increased the awareness of clinicians about the subject.

## Introduction

The famous scientist Galileo Galilei, who is known as the father of modern physics, once said “Doubt is the father of invention” (*1*). He emphasized that the most important factor triggering the development of science was doubt. It is obvious that every subject that is suspected and therefore investigated is more open to development and progress. The word uncertainty means doubt. In its broadest definition, measurement uncertainty means doubt about the validity of the measurement results (*2*).

Today, in economics, physics, chemistry and many other fields the concepts of doubt and uncertainty find a larger area day by day because every result of a measurement comes with the “uncertainty” arising from its own nature (*3*-*5*).

Measurements made in clinical laboratories play an important role in diagnosis, treatment and follow-up of diseases. However, it is known that when any test is repeated, even if all conditions are optimized, the probability of achieving exactly the same result is very low. In other words, the results of chemical reactions are actually components of a distribution. At this point, it would be a proper approach to talk about the measurement uncertainty of results.

According to the definition of the International Vocabulary of Metrology, measurement uncertainty (MU) is non-negative parameter characterizing the dispersion of the quantity values being attributed to a measurand, based on the information used (*6*). In simple terms, it is a quantitative indicator of the range in which the “real” result may actually be.

In laboratory measurements, there are many variables related to sampling, transport and storage, preparation of reagents, maintenance of devices, measurement method *etc.* In order to obtain reliable results, sources causing variation should be identified and minimized (*7*). The International Organization for Standardization (ISO) 15189 guide, which prepared for the standardization of medical laboratories, requires laboratories to have a defined method for establishing the MU of tests, determining performance criteria, and reviewing MU data regularly (*8*).

Generally, two approaches can be mentioned about the MU calculations. These are bottom-up and top-down approaches (*9*). The top-down approach prioritizes the use of quality control results for calculations. Random and systematic errors need to be resolved for this purpose. In the mathematical expression of these errors; internal quality control data is used for random errors and external quality assessment (EQA) can be used for systematic errors. This approach makes it very easy to adapt applications to clinical laboratories. According to the bottom-up approach, all uncertainty components are rigorously detected. All of the above-mentioned variables are examined one by one and included in the calculation in proportion to their contribution to uncertainty. That process is considered laborious for the clinical laboratories. Moreover, such complex calculations may not be necessary, given that the effects of any uncertainty components are reflected in the results of the internal control sample studied daily just like a patient sample. Since similar results were found in a study in which uncertainty calculations were made according to both approaches, it was recommended to use the top-down approach as a simpler method (*10*).

There is no restriction about which method to use. Unfortunately, there is no internationally recognized guide to compare whether the results are appropriate or not. Therefore it is important to publish the calculated MU of tests for comparison between laboratories.

When the literature was researched, for drug concentration tests no study was found using same/similar method. In this respect, we believe that our study will shed light on future research. The purpose of this study is to present an example of calculating MU that is practical to use in clinical routine and to discuss how the results can be evaluated.

## Materials and methods

In our study, it was planned to calculate the MU of immunoassay tests like fertility hormones, drug concentration tests, cardiac markers, thyroid function tests and tumour markers using the internal quality control results and EQA data of our laboratory between January 01, 2018 and December 31, 2018. Permission was obtained from the Ethics Committee of Aydın Adnan Menderes University Faculty of Medicine Non-Invasive Clinical Research with protocol number 2020/09. Table 1 shows the tests included to the study and their measurement methods with ranges.

**Table 1:** Methods and measurement ranges of tests

**Test**	**Method**	**Range**
βHCG	CMIA	1.2-15,000 mIU/mL
DHEAS	CMIA	3-1500 μg/dL
FSH	CMIA	0.05-150.00 mIU/mL
LH	CMIA	0.09-250.00 mIU/mL
E_2_	CMIA	10-1000 pg/mL
PROG	CMIA	0.1-40.0 ng/mL
PROL	CMIA	0.6-200.0 ng/mL
TEST	CMIA	0.15-64.57 nmol/L
DIG	CMIA	0.3-4.0 ng/mL
PHNY	Enzyme immunoassay	1.8-40.0 μg/mL
PHNO	PETINIA	2-80 μg/mL
CARB	PETINIA	1.9-20.0 μg/mL
VPA	PETINIA	2-150 μg/mL
CK-MB	Immune inhibition	0.1–300.0 ng/mL
MYG	CMIA	1-1200 ng/mL
TnI	CMIA	3.2–50,000 pg/mL
fT3	CMIA	1-30 pg/mL
fT4	CMIA	0.4-5.0 ng/dL
TSH	CMIA	0.0025-100 μIU/mL
AFP	CMIA	10-2000 ng/mL
CA15-3	CMIA	0.5-800.0 U/mL
CA19-9	CMIA	2-1200 U/mL
CA 125	CMIA	1-1000 U/mL
CEA	CMIA	0.5-1500.0 ng/mL
fPSA	CMIA	0.008-30 ng/mL
tPSA	CMIA	0.008-100 ng/mL
βHCG - human chorionic gonadotropin. DHEAS - dehydroepiandrosterone sulfate. FSH - follicle stimulating hormone. LH - luteinizing hormone. E_2_ – estradiol. PROG – progesterone. PROL – prolactin. TEST – testosterone. DIG – digoxin. PHNY – phenytoin. PHNO - phenobarbital, CARB – carbamazepine. VPA - valproic acid. CK-MB - creatine kinase myocardial band. TnI - troponin-I. MYG – myoglobin. fT3 - free triiodothyronine. fT4 - free thyroxine. TSH - thyroid stimulating hormone. AFP - alpha-fetoprotein. CA15-3 - cancer antigen 15-3. CA19-9 - cancer antigen 19-9. CA 125 - cancer antigen 125. CEA - carcinoembryonic antigen. fPSA - free prostate specific antigen. tPSA - total prostate specific antigen. CMIA - Chemiluminescent Microparticle Immunoassay. PETINIA - Particle-enhanced Turbidimetric Inhibition Immunoassay.		

Parameters of drug concentration tests were performed on the Abbott Architect c8000 (Abbott Diagnostics, Abbott Park, USA), and the remaining hormones and biomarkers were studied on the Abbott Architect i2000 SR hormone analyser using the company’s original kits and calibrators. Internal quality control studies were carried out daily and external quality control studies were performed once a month. The EQA goal of our laboratory is to prevent the z-score from rising above 3, and try to keep it below 2 as much as possible. The z-score is a measure of the laboratory’s bias relative to comparator group. A z-score ≤ 2 is acceptable; if it is 2-3, a warning and an investigation are recommended; a z-score is ≥ 3 is unacceptable and remedial action usually is required. The EQA data of every parameter was compared with its peer group except drug concentration tests. Because of the number of equivalent group laboratories was low (< 20), the calculations for drugs were made according to the mode group or overall results.

## Calculation steps

The top-down approach was chosen. For this purpose the steps of the Nordtest guide were followed (*11*):

1. Specify measurand, range, and target MU.
2. Quantify standard uncertainty component for the within-laboratory reproducibility (u(Rw)).
3. Quantify the uncertainty component associated with method and laboratory bias (u(bias)).
4. Convert components to standard uncertainty (u(x)).
5. Calculate combined standard uncertainty (u_c_).
6. Calculate expanded uncertainty (U=k x u_c_. where k = 2 (95% confidence interval)).

The standard uncertainty component for the within-laboratory reproducibility (u(Rw)) is derived from internal control sample results collected long enough to cover all worst-case scenarios and studied just like a patient sample. The number of results should ideally be more than 60 and cover a time period of at least one year to reflect all variations such as different stock solutions, new batches of critical reagents, recalibrations of equipment (*11*).

Internal quality control results were examined separately according to the lots of each level. Coefficient of variation (CV%) is the standard deviation expressed as a percentage of the mean. The CV is useful because it is independent of concentration. Coefficient of variation is calculated from standard deviation (SD) and mean of internal control sample results (CV% = (SD/Mean)x100). Then, the standard uncertainty component for the within-laboratory reproducibility is calculated as u(Rw) = Rw/2 where Rw = (Σ(CV%)^2^/N))^1/2^ and N is the number of calculated CV%.

For method and laboratory bias (u(bias)), two bias components have to be estimated: 1) the root mean square of the individual bias values (RMS_bias_), and 2) the mean of the standard uncertainty of the assigned values (u(Cref)). There are three ways to calculate u(bias) component, namely; the use of Certified Reference Material, participation in EQA and performing recovery tests. Use of EQA was the most practical and cost effective way. However, for reference materials a mean value over time is used and for each EQA a single laboratory result is used. Therefore the estimated RMS_bias_ from EQA will usually be higher. In order to have a reasonably clear picture of the bias from EQA results, a laboratory should participate at least 6 times within a reasonable time interval (*11*). The calculations are as follows: RMS_bias_ = (Σ(bias)^2^/N))^1/2^, where N = number of EQA rounds; u(Cref) = Σ(CV_EQA_/N_Lab_^1/2^)^2^/N, where N_Lab_ = number of participating laboratories in each round and CV_EQA_ is the CV% of each EQA round.

The standard uncertainty (u(x)) is calculated as follows: u(bias) = (RMS_bias_^2^ + u(Cref)^2^)^1/2^. Combined standard uncertainty (u_c_) equals u_c_ = (u(Rw)^2^ + u(bias)^2^)^1/2^, while expanded uncertainty U = 2xu_c_.

Since there are no universally accepted limits that determine the measurement uncertainty of the tests, it was planned to make a comparison according to the allowable total error and biological variation data, like many other articles (*12*-*14*).

## Results

In order to make these complex calculations more descriptive, the example of calculation for a parameter is demonstrated. Table 2 shows the calculations for u(Rw) component of phenytoin (PHNY). Table 3 presents the one-year EQA results for PHNY. CV_EQA_ and Bias values in that table are provided by the EQA program server.

**Table 2:** Calculations for standard uncertainty component for the within-laboratory reproducibility of phenytoin

**N**	**Level**	**Lot number**	**Mean**	**SD**	**CV (%)**	**Rw (%)**	**u(Rw) (%)**
**107**	1	17205170	6.9 μg/mL	0.5 μg/mL	7.7	8.1	4.1
**52**	1	17610170	7.7 μg/mL	0.6 μg/mL	8.1		
**110**	2	17205170	12.0 μg/mL	1.0 μg/mL	8.1		
**57**	2	17610170	13.0 μg/mL	1.1 μg/mL	8.5		
SD - standard deviation. CV - coefficient of variation. Rw - Run within-laboratory. u(Rw) -standard uncertainty component for the within-laboratory reproducibility.							

**Table 3:** One-year external quality control results of the phenytoin test

**Month**	**Result**	**Target**	**N_Lab_**	**Bias (%)**	**CV_EQA_(%)**	**z-score**
January	31.9 μg/mL	30.5 μg/mL	132	4.4	8.4	0.5
February	20 μg/mL	18 μg/mL	186	10	11	1.2
March	6.5 μg/mL	5.65 μg/mL	186	15	9.9	1.5
April	12.4 μg/mL	11.8 μg/mL	203	4.9	8.1	0.6
May	19 μg/mL	18.1 μg/mL	211	4.7	9.1	0.5
June	4.8 μg/mL	5.6 μg/mL	207	14	8.6	1.6
July	38.4 μg/mL	30.7 μg/mL	200	25	8.3	2.9
August	14.2 μg/mL	11.9 μg/mL	213	19	8.8	2.1
September	6 μg/mL	5.68 μg/mL	207	5.6	8.4	0.6
October	21.2 μg/mL	18.2 μg/mL	202	16	9.9	1.6
November	12.2 μg/mL	12 μg/mL	211	1.9	8.7	0.2
December	33.7 μg/mL	30.7 μg/mL	201	9.9	9.3	1
N_Lab_ - number of participating laboratories in each round. Bias - percentage deviation from the assigned value. CV_EQA_ - coefficient of variation of each external quality assessment round. z-score - number of standard deviations that your result differs from the assigned value.						

According to the Nordtest approach, calculation steps for all parameters are shown in Table 4. For each test, u(bias) value was higher than u(Rw) value. The volume of u(Cref) affected the MU more minimally than the others. Prolactin had the lowest MU (8.3%) and CA 19-9 had the highest MU (28%).

**Table 4:** Calculated standard uncertainty component for the within-laboratory reproducibility, method and laboratory bias, combined standard uncertainty and expanded uncertainty for each parameter according to the Nordtest guide

	**u(Rw)**	**u(Cref)**	**RMS_bias_**	**u(bias)**	**u_c_**	**U(%)**
βHCG	4.7	0.2	8.1	8.1	9.3	19
DHEAS	2.2	0.4	6.8	6.8	7.2	14
FSH	1.8	0.3	4.2	4.2	4.6	9.3
LH	2.6	0.4	5.4	5.4	6.1	12
E_2_	2.2	0.3	7.6	7.6	7.9	16
PROG	3.1	0.4	5.4	5.4	6.3	13
PROL	1.7	0.4	3.7	3.8	4.1	8.3
TEST	1.7	0.7	6	6.1	6.3	13
DIG	4.4	0.6	5.9	5.9	7.4	15
PHNY	4	0.6	12.9	12.9	13	27
PHNO	2.9	0.7	6.3	6.4	7	14
CARB	3.9	2.6	9.9	10.0	11.0	22
VPA	2.7	1.1	5.3	5.5	6.1	12
CK-MB	3.3	1.3	6.6	6.7	7.5	15
MYG	3.2	1.3	6.3	6.4	7.2	14
TnI	4.1	1.4	8.2	8.3	9.3	19
fT3	2.8	0.3	8.3	8.3	8.8	18
fT4	2.5	0.3	5.8	5.8	6.4	13
TSH	1.9	0.2	7	7	7.2	15
AFP	1.7	0.3	4.3	4.3	4.7	9.4
CA15-3	3.4	0.4	7.2	7.2	8	16
CA19-9	5.3	0.6	12	12	14	28
CA 125	2.4	0.3	4.8	4.9	5.4	11
CEA	2.5	0.3	5.9	5.9	6.4	13
fPSA	2	0.3	5.3	5.3	5.6	11
tPSA	2.8	0.3	6	6.1	6	13
u(Rw) - standard uncertainty component for the within-laboratory reproducibility. u(Cref) - the mean of the standard uncertainty of the assigned values. RMS_bias_ - the root mean square of the individual bias values. u(bias) - method and laboratory bias. u_c_ - combined standard uncertainty. U - expanded uncertainty. βHCG - human chorionic gonadotropin. DHEAS - dehydroepiandrosterone sulfate. FSH - follicle stimulating hormone. LH - luteinizing hormone. E_2_ – estradiol. PROG – progesterone. PROL – prolactin. TEST – testosterone. DIG – digoxin. PHNY – phenytoin. PHNO - phenobarbital. CARB – carbamazepine. VPA - valproic acid. CK-MB - creatine kinase myocardial band. TnI - troponin-I. MYG – myoglobin. fT3 - free triiodothyronine. fT4 - free thyroxine. TSH - thyroid stimulating hormone. AFP - alpha-fetoprotein. CA15-3 - cancer antigen 15-3. CA19-9 - cancer antigen 19-9. CA 125 - cancer antigen 125. CEA - carcinoembryonic antigen. fPSA - free prostate specific antigen. tPSA - total prostate specific antigen.						

Table 5 presents calculated MU results of tests and allowable performance limits of various guidelines (*15*-*20*). According to that table, 12 of the 26 parameters (follicle stimulating hormone (FSH), luteinizing hormone (LH), progesterone (PROG), Prolactin (PROL), testosterone (TEST), creatine kinase myocardial band (CK-MB), myoglobin (MYG), troponin-I (TnI), thyroid stimulating hormone (TSH), alpha-fetoprotein (AFP), cancer antigen 125 (CA 125), free prostate specific antigen (fPSA)) have uncertainty values below the limits recommended by all of the mentioned guidelines. Excluding RCPA recommendations, which seem to offer a narrower range than other guidelines, human chorionic gonadotropin (βHCG), digoxin (DIG), phenobarbital (PHNO), valproic acid (VPA), cancer antigen 15-3 (CA 15-3), cancer antigen 19-9 (CA 19-9), carcinoembryonic antigen (CEA) and total prostate specific antigen (tPSA) also met all of the recommendations. Two parameters (dehydroepiandrosterone sulfate (DHEAS) and PHNY) are labelled as ‘Failed’ according to all lists.

**Table 5:** Comparison of calculated measurement uncertainty results and allowable performance limits

	**U(%)**	**Westgard (%)**	**CLIA (%)**	**Ricos (%)**	**Rili-BAEK (%)**	**RCPA (%)**	**EFLM (%)**
βHCG	19	/	/	/	30	10	/
DHEAS	14	13.8	/	11.5	/	12	/
FSH	9.3	21.19	/	20.2	21	10	42.1
LH	12	27.92	/	24.7	/	15	30.8
E_2_	16	26.86	/	34.6	35	25	13
PROG	13	/	/	/	35	15	/
PROL	8.3	29.4	/	23.4	/	10	43
TEST	13	13.61	/	16	35	15	21.4
DIG	15	/	20	/	30	10	/
PHNY	27	/	25	/	20	10	/
PHNO	14	/	20	/	20	10	/
CARB	22	/	25	/	20	10	/
VPA	12	/	25	/	20	10	/
CK-MB	15	30	/	37.4	/	20	/
MYG	14	19.6	/	24.4	/	/	/
TnI	19	27.91	/	/	33	20	35.9
fT3	18	9.22	/	14.3	20	20	8.3
fT4	13	8	20	10.4	20	12	7.8
TSH	15	23.7	/	31.3	24	20	35.9
AFP	9.4	21.9	/	/	24	12	55.2
CA15-3	16	20.8	/	17.5	24	10	/
CA19-9	28	46.3	/	52.6	/	15	57.4
CA 125	11	35.4	/	28	/	12	25.5
CEA	13	24.7	/	24.9	24	12	59.3
fPSA	11	/	/	/	/	15	46.2
tPSA	13	33.6	/	34.7	25	8	42
U - calculated uncertainty results of tests. Westgard - Westgard’s desirable specification for allowable total error (15). CLIA - Clinical Laboratory Improvement Amendments, Criteria for acceptable analytical performance (16). Ricos - Ricos’s databases on biologic variation (17). Rili-BAEK - German Guidelines for Quality, Acceptable relative deviation in interlab tests (18). RCPA - Royal College of Pathologists of Australasia, Allowable limits of performance for biochemistry (19). EFLM - European Federation of Clinical Chemistry and Laboratory Medicine Biological Variation Database (20). βHCG - human chorionic gonadotropin. DHEAS - dehydroepiandrosterone sulfate. FSH - follicle stimulating hormone. LH - luteinizing hormone. E_2_ – estradiol. PROG – progesterone. PROL – prolactin. TEST – testosterone. DIG – digoxin. PHNY – phenytoin. PHNO - phenobarbital. CARB – carbamazepine. VPA - valproic acid. CK-MB - creatine kinase myocardial band. TnI - troponin-I. MYG – myoglobin. fT3 - free triiodothyronine. fT4 - free thyroxine. TSH - thyroid stimulating hormone. AFP - alpha-fetoprotein. CA15-3 - cancer antigen 15-3. CA19-9 - cancer antigen 19-9. CA 125 - cancer antigen 125. CEA - carcinoembryonic antigen. fPSA - free prostate specific antigen. tPSA - total prostate specific antigen.							

## Discussion

Determining MU from EQA data is practical and cost effective but can greatly increase the MU. Similarly, the RMS_bias_ values which calculated from the EQA results, were the most important parameter that influences the MU. Table 4 demonstrates that impact apparently. For example about PHNY, if we ignore the July EQA evaluation, which has the highest bias, the MU of PHNY would have decreased from 27% to 24%, and it would have been below the recommendation 25% determined by CLIA. From this point of view, it is seen that only one mistake made during the PT programs can affect the whole results. It is obvious that detailed arrangements are needed in this regard. Secondly it can be recommended that if RMS_bias_ or u(Cref) values are higher than a certain value, these components may not be suitable for such calculations.

The ‘Failed’ test, PHNY, is evaluated intently. There was no study that could be observed for result comparison. The largest share of the uncertainty components was the u(bias) value. However, according to Table 3 even in July, the EQA period with the highest bias for PHNY, the z-score was 2.99. Even when the internal and external quality results of a parameter seem within limits, the MU of that test may be higher than desired. For this reason, MU results should be followed closely, just like internal and external quality control results.

When our other ‘Failed’ test, DHEAS, was examined, it was seen that the uncertainty value found was not far beyond the limits specified by the guidelines. The MU of the parameter was found to be 14% for our laboratory. The recommendations of Westgard, Ricos and RCPA are 13.8%, 11.5%, 12%, respectively. However, to improve the parameter, all PT data were re-examined for u(bias), which has the dominant factor on the increasing of MU, it was noticed that none of the 12 participants to EQA had a z-score above two. At this point, it was thought that MU calculations could guide us in recognizing overlooked problems.

In a study conducted by Ayyıldız, the MU for DHEAS was reported as 15.5% (*21*). When that report was examined, it was seen that the same brand and model device was used as in our study. The presence of similar results for DHEAS at the same device may suggest that the method or device performance should be improved. It would be useful to compare method performances by performing similar studies with other brand devices. Similarly, if high MU data is obtained for DHEAS, the target value may tend to increase.

For cardiac markers; CK-MB, MYG and TnI, it can be said that the limits of all lists are complied with and no corrective action is required. The same is true for the TSH parameter. However, the uncertainties of fT3 (18%) and fT4 (13%) tests exceeded the limits of some guidelines, although they were appropriate according to others. In such cases, the laboratory specialist should interpret results based on his/her experiences. In a study by Çubukçu et al., the MU of fT3 and fT4 was calculated according to a similar approach and reported as 15.92% and 24.04%, respectively (*22*).

It has been observed that three tumour markers (AFP, CA 125 and fPSA) are appropriate for all lists, and the remaining parameters do not exceed the limits of guidelines, except for the RCPA recommendations.

The high permissible quality limits recommended by the guidelines for CA 19-9 are noteworthy. It was recommended by Westgard, Ricos and EFLM 46.3%, 52.6% and 57.4%, respectively. The variation value, up to half of the given result, should lead laboratory experts to be more attentive about results of this parameter. Particular attention should be paid to the comparison of results produced by different laboratories, and clinicians should be warned about that when necessary.

There is no standardized formula for clinical laboratories to estimate MU of tests (*12*). Furthermore there is no standardized limits to compare results. Thus every laboratory should define its own formulation system and evaluation method. And so errors can be noticed by renewing calculations at certain periods.

Measurement uncertainty is among the standards as a criterion in quality assessment. The fact remains that, such calculations will not become prevalent unless they are practically done in daily routine applications. Hence MU models that can be calculated using the available data without the need for a separate budget are important and valuable.

The limitation of this study is the lack of possibility to calculate EQA data according to our peer group for drug concentration tests. Although we predict that this situation will increase the uncertainty value of the parameters calculated from the EQA data, we think that special attention should be paid to the measurement uncertainty calculations of these tests, especially since they are analytes that must be kept in a narrow range in plasma and must be precisely adjusted.

In conclusion, our study showed that there can be a wide range of recommendations for a parameter and the importance of choosing which guideline to follow. A test that is appropriate according to one guideline may give very poor results according to another.

It is recommended that the concept of measurement uncertainty, which plays an important role in the total quality performance of the laboratory, should be followed up by the clinical laboratory experts at certain time intervals and should be increased the awareness of clinicians about the subject.
